# Insight into Details of the Photosynthetic Light Reactions and Selected Metabolic Changes in Tomato Seedlings Growing under Various Light Spectra

**DOI:** 10.3390/ijms222111517

**Published:** 2021-10-26

**Authors:** Monika Kula-Maximenko, Ewa Niewiadomska, Anna Maksymowicz, Agnieszka Ostrowska, Jana Oklestkova, Aleš Pěnčík, Anna Janeczko

**Affiliations:** 1 Polish Academy of Sciences, The Franciszek Górski Institute of Plant Physiology, Niezapominajek 21, 30-239 Cracow, Poland; m.kula@ifr-pan.edu.pl (M.K.-M.); e.niewiadomska@ifr-pan.edu.pl (E.N.); a.maksymowicz@ifr-pan.edu.pl (A.M.); a.ostrowska@ifr-pan.edu.pl (A.O.); 2 Laboratory of Growth Regulators, Faculty of Science and Institute of Experimental Botany of the Czech Academy of Sciences, Palacký University, Šlechtitelů 27, CZ-78371 Olomouc, Czech Republic; jana.oklestkova@upol.cz (J.O.); pencik@ueb.cas.cz (A.P.)

**Keywords:** auxins, brassinosteroids, plant growth, photosynthesis, light spectral composition, *Solanum lycopersicum* L.

## Abstract

The objective of our study was to characterise the growth of tomato seedlings under various light spectra, but special attention has been paid to gaining a deeper insight into the details of photosynthetic light reactions. The following light combinations (generated by LEDs, constant light intensity at 300 μmol m^−2^ s^−1^) were used: blue/red light; blue/red light + far red; blue/red light + UV; white light that was supplemented with green, and white light that was supplemented with blue. Moreover, two combinations of white light for which the light intensity was changed by imitating the sunrise, sunset, and moon were also tested. The reference point was also light generated by high pressure sodium lamps (HPS). Plant growth/morphological parameters under various light conditions were only partly correlated with the photosynthetic efficiency of PSI and PSII. Illumination with blue/red as the main components had a negative effect on the functioning of PSII compared to the white light and HPS-generated light. On the other hand, the functioning of PSI was especially negatively affected under the blue/red light that was supplemented with FR. The FT-Raman studies showed that the general metabolic profile of the leaves (especially proteins and β-carotene) was similar in the plants that were grown under the HPS and under the LED-generated white light for which the light intensity changed during a day. The effect of various light conditions on the leaf hormonal balance (auxins, brassinosteroids) is also discussed.

## 1. Introduction

Tomato (*Solanum lycopersicum* L.) is a species of the family *Solanaceae* that originated in South America. In Poland and other countries that have a colder climate, in the case of undercover productions, additional lighting has to be used for tomato production, which increases the costs—this applies to production for fruit yield, but also for the production of seedlings. High-pressure sodium lamps (HPS), which emit large amounts of heat, are still used to illuminate most of the tomato crops that are grown in greenhouses in Poland. This is mainly because, according to many producers, the growth and yield of tomatoes under commercial light-emitting diode (LED) modules are less satisfactory than for those that are grown under the standard HPS lighting. This has generated the need for further research whose aim is to optimise lighting conditions (the light spectrum, its intensity, light modulation) in order to improve growth/yield while simultaneously taking into account the specificity of a particular cultivar.

Although the range of visible light includes a wavelength of 380–760 nm, the strongest absorption of radiation by plants lies in the blue (460 nm) and red (660 nm) ranges due to their photosynthetic pigments, primarily from the group of chlorophylls. 

However, the two photosystems PSII and PSI have distinct light-harvesting properties with maximum absorption at 680 nm and 700 nm, respectively [1]. Following the action spectra of PSII and PSI in the entire range of PAR (400–700 nm) also revealed some other differences [2]. Hence, changes in the spectrum of growth light may lead to overexcitation of one of the photosystems and to imbalance in the linear electron flow. Such a situation is counteracted by several strategies which optimize the photosynthetic electron flux through both photosystems, among them: fine tuning of PSII antenna, the number of PSI units, state transition, cyclic electron transfer, and many others [3,4].

For a long time, it was thought that red and blue light had the greatest impact on plant growth and were the main energy source for CO_2_ fixation during photosynthesis [5]. Red light is, among others, essential for the development of the photosynthetic apparatus [6,7], while blue light affects the biosynthesis of chlorophyll, the development of the chloroplasts and the movement of the stomata and also participates in photomorphogenesis [8,9,10]. Therefore, because plants absorb mainly blue and red light for photosynthesis, light with a narrow blue+red spectral composition that was supplemented with LED light sources was initially recommended for greenhouse cultivation. It was proven that lighting in these narrow bands could also affect the weight of a plant and the content of various organic compounds, including antioxidants [11]. However, more and more research results began to indicate that different species and even different cultivars of plants require a broader spectrum of light for optimal growth and can also have a different reaction to the same spectral composition of light [12]. To date, four groups of photoreceptors, which sense different parts of the light spectrum, have been characterised in plants [13]. Phytochrome is sensitive to light between the red and far-red regions of the spectrum (600–750 nm). Cryptochrome and phototropin absorb in the regions of blue and UVA light (320–500 nm). There is also a less described receptor that absorbs light in the UVB region (282–320 nm). It is possible that other photoreceptors still remain undiscovered. Photoreceptors are used by plants to obtain information about the quantity of light and the duration (periodicity) of irradiation, which consequently influence many metabolic reactions [13,14,15]. A particular plant species might also require spectral composition of light to be selected and adjusted for use in greenhouse production [16,17]. Although LED producers were rather focused on blue/red light in the beginning, the addition of green light to the spectrum of LED lighting has been attracting more and more attention. It is known from previous studies that it is important in many of the physiological processes in plants ([18,19] and literature cited in these works). According to Sun at al. [18], already in 1928, it was revealed that green light is active in the production of chlorophyll. The green wavebands, among others, induce stem growth elongation and adjust the plastid transcriptome during early photomorphogenic development in Arabidopsis [20]. In lettuce, green supplementation enhanced plant growth under red and blue light-emitting diodes [21].

Although it is known that it is possible to cultivate tomatoes under LED lamps [22,23,24,25,26,27], new discoveries in this area are still being reported in the literature that could further help to optimise this technology for this species. In the studies of Dieleman et al. [28], young tomato plants were cultured under blue, green, amber, red, white, or red/blue LED light with a low intensity background (sunlight). Under blue light, the plants were shorter with smaller leaves but had the highest levels of light-harvesting pigments, although this was not accompanied by a high rate of leaf photosynthesis. Under green light, the tomato plants were taller but had more horizontally oriented leaves that enabled a higher degree of light transmission deeper into the canopy. Additionally, the authors observed that the highest rate of photosynthesis was under red light, but only in plants that had initially been grown under green light. Similarly, plants that had been grown under blue light had a low rate of photosynthesis, but after exposure to white light, these leaves had the highest rate of photosynthesis. According to the authors, dynamic light spectra create opportunities to increase growth and production in systems such as a greenhouse or vertical farming. On the other hand, Kaiser et al. [29] found that in tomatoes, biomass and yield was stimulated by a partial replacement of red and blue light with green light. Green light increased the leaf and stem biomass, as well as the leaf area and the carotenoid concentration, which was also higher in the canopy. Recently, Paponov et al. [30] found that supplemental LED inter-lighting (80% red, 20% blue) to the HPS lamps also enhanced the growth of tomato plants and increased the weight of the fruits. Palmitessa et al. [31] studied the effect of light intensity on tomato growth and yield and found that too-long continuous lighting caused the formation of leaf chlorosis. PPFD of more than 500 μmol m^−2^ s^−1^ caused leaf stress and physiological disorders and increasing light intensity reduced gas exchange.

The objective of our study was to characterise the impact of various spectral compositions of light on growth of tomato seedlings, with special attention being paid to gaining a deeper insight into the details of photosynthetic light (and dark reactions). Specifically, the efficiency of the reactions was analysed in regard to the orchestrated functioning of PSI and PII in tomato plants that were grown under two main groups of lighting—blue/red light variants and white light variants, including white light combinations in which the sunrise, sunset, and moon were imitated by changing the light intensity. The effect of various light conditions on the leaf metabolic profile (measured using FT-Raman spectroscopy) and the balance of the growth hormones (auxins, brassinosteroids) is also discussed.

## 2. Results and Discussion

### 2.1. Plant Growth

**Figure 1:**
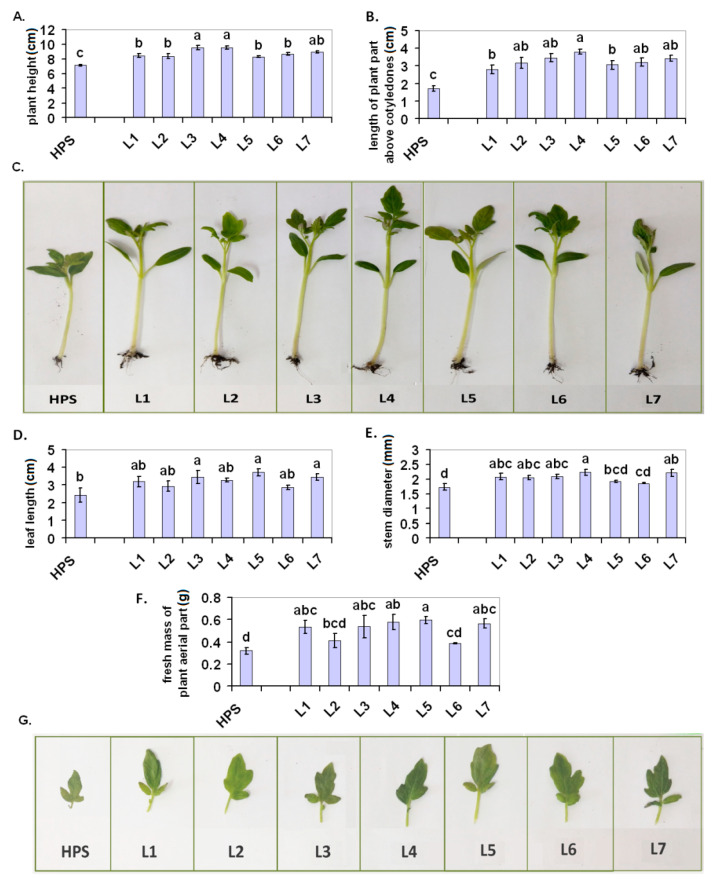
Plant growth/morphological parameters of the 12-d-old seedlings of the tomatoes that were grown under the HPS lamps and under various LED light spectra (L1–L7). (**A**) Plant height, (**B**) length of the plant aerial part above the cotyledons, (**C**) exemplary 12-d-old seedlings, (**D**) first leaf length, (**E**) stem diameter under the cotyledons, (**F**) fresh mass of the arterial part of the plants, (**G**) exemplary first leaves. Values (±SD) marked with the same letters are not significantly different according to the Duncan test (*p* ≤ 0.05). HPS—sodium lamp; L1—blue/red light; L2—blue/red light + far red; L3—blue/red light + UV; L4—white light supplemented with green; L5—white light supplemented with blue; L6—white light supplemented with red; L7—white light supplemented with blue/UV. HPS—L5—constant light 300 μmol m^−2^ s^−1^; L6–L7 light modulated (sunrise and sunset accomplished by gradually increasing/decreasing the light intensity, in the middle of the day the light intensity was increased to 700 μmol m^−2^ s^−1^ for 2 h; for details see chapter 3.1).

**Figure 2:**
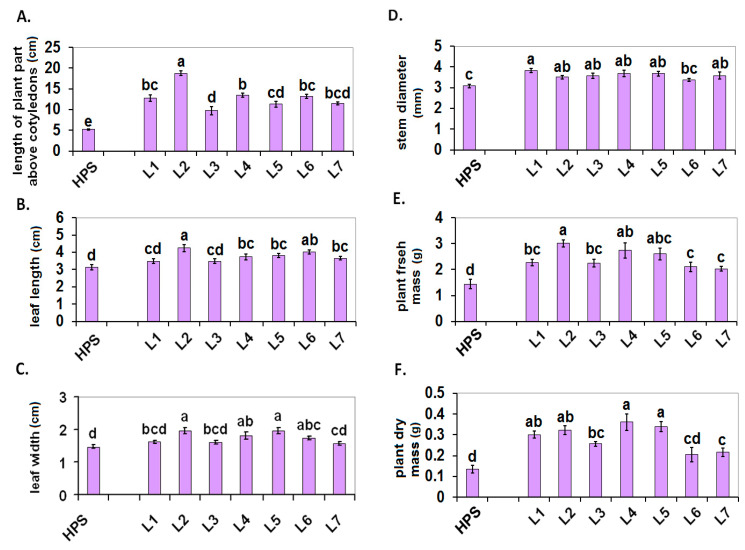
Plant growth/morphological parameters of the 30-d-old plants of the tomatoes that were grown under the HPS lamp and under various LED light spectra (L1–L7). (**A**) Length of the aerial part of the plant above the cotyledons, (**B**) fourth leaf length, (**C**) fourth leaf width (**D**) stem diameter under the cotyledons, (**E**) fresh mass of the aerial part of the plants, (**F**) dry mass of the aerial part of the plants. Values (±SD) marked with the same letters are not significantly different according to the Duncan test (*p* ≤ 0.05). HPS—sodium lamp; L1—blue/red light; L2—blue/red light + far red; L3—blue/red light + UV; L4—white light supplemented with green; L5—white light supplemented with blue; L6—white light supplemented with red; L7—white light supplemented with blue/UV. HPS—L5—constant light 300 μmol m^−2^ s^−1^; L6-L7 light modulated (sunrise and sunset accomplished by gradually increasing/decreasing the light intensity, in the middle of the day the light intensity was increased to 700 μmol m^−2^ s^−1^ for 2 h; for details see chapter 3.1).

The 12-d-old tomato seedlings that were grown under the LED lamps were more elongated than the seedlings that were grown under the HPS. This was visible in the plant height, which was measured from the base of the stem and also when it was only measured above the cotyledons (Figure 1A,B, photo in Figure 1C). All of the seedlings that were grown under the LED lamps had longer leaves than the plants that were grown under the HPS, although a statistically significant difference was only proven for L3, L5, and L7 (Figure 1D). The photo (Figure 1G) shows exemplary leaves from HPS and L1–7. Only the seedlings from L1 to L4 and L7 had a larger stem diameter than the seedlings from the HPS (Figure 1E). All of the plants that were grown under the LED lamps showed higher accumulation of fresh weight than the seedlings that were grown under the HPS, although no statistically significant difference was proven for L2 and L6 (Figure 1F). For the 30-d-old plants (Figure 2A–F), the values of all of the growth/morphological parameters that were measured were higher in the plants that were grown under the LED lamps (although in some cases, no statistically significant difference was proven and only a trend was observed). The architecture of exemplary 30-d-old plants is shown in Figure S2 (Supplementary Materials).

According to Kaiser et al. [32], appropriately selected supplementation with red and blue LED light could be beneficial for tomato growth in artificial/indoor cultivation, which was also visible in our experiment. On the other hand, UVA radiation could also stimulate the growth of tomatoes via a morphological adaptation, which would lead to an increased light interception [33]. In our experiment, the addition of UV, i.e., for L7 compared to L6 had some rather slight effects on plant growth, which was visible only in the younger (12-d-old) seedlings (Figure 1D–F). One of the most important indicators of light efficiency seems to be the dry mass accumulation (Figure 2F), which was significantly increased under the LED lamps compared to the HPS, and L4 and L5 light worked particularly well. In both of these cases, green light had a relatively large share (especially at L4). This is consistent with earlier observations by Dieleman et al. [28] and Kaiser et al. [29], who also found a beneficial effect of green light on tomato growth stimulation and biomass accumulation.

### 2.2. Photosynthesis

The efficiency of the photosynthetic apparatus is crucial for the productivity of crop plants. Therefore, in the presented study, we analysed the photosynthetic efficiency of photosynthesis using several approaches such as (1) the condition of photosystem II in the dark-adapted state, (2) the quantum efficiencies of photosystems I and II during light adaptation, and (3) the intensity of the leaf–gas exchange.

**Figure 3:**
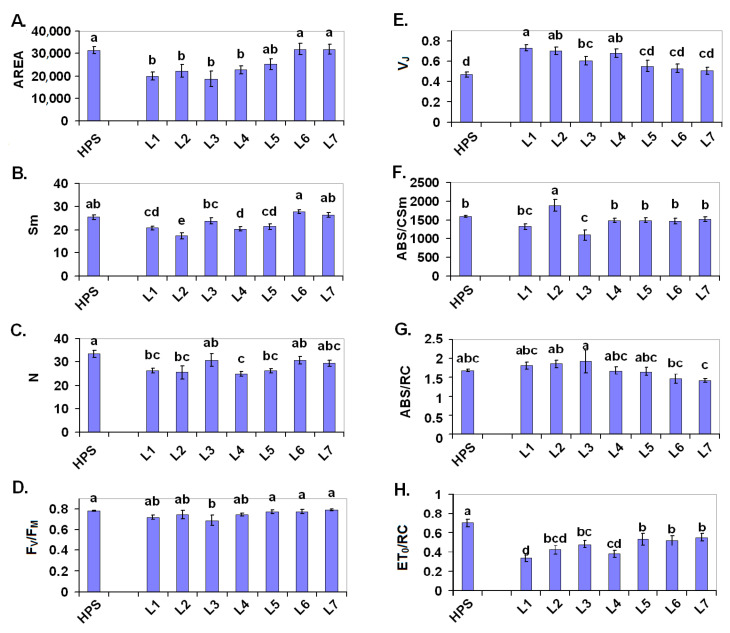
OJIP test parameters in the 30-d-old seedlings of the tomatoes that were grown under the HPS lamp and under various LED light spectra (L1-L7): AREA (**A**), Sm (**B**), N (**C**), F_V_/F_M_ (**D**), V_J_ (**E**), ABS/CSm (**F**), ABS/RC (**G**), ET_0_/RC (**H**). Values (±SD) marked with the same letters are not significantly different according to the Duncan test (*p* ≤ 0.05). HPS—sodium lamp; L1—blue/red light; L2—blue/red light + far red; L3—blue/red light + UV; L4—white light supplemented with green; L5—white light supplemented with blue; L6—white light supplemented with red; L7—white light supplemented with blue/UV. HPS—L5—constant light 300 μmol m^−2^ s^−1^; L6–L7 light modulated (sunrise and sunset accomplished by gradually increasing/decreasing the light intensity, in the middle of the day the light intensity was increased to 700 μmol m^−2^ s^−1^ for 2 h; for details see chapter 3.1).

**Figure 4:**
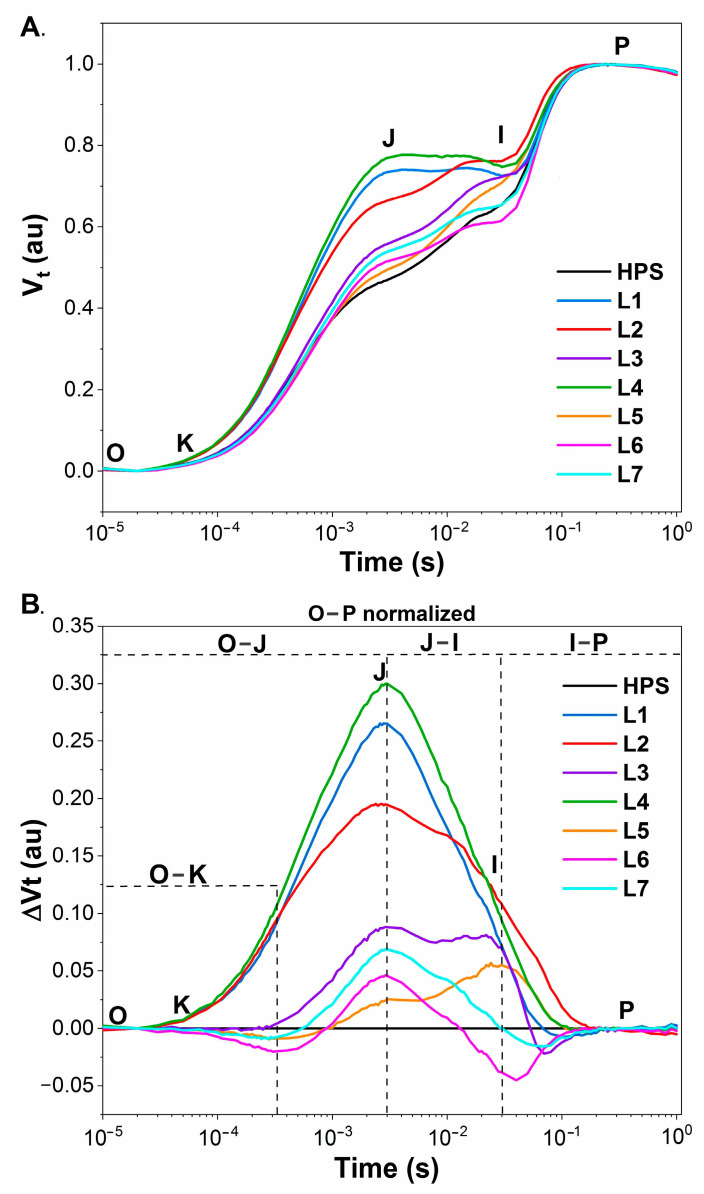
The chlorophyll fluorescence transients from the dark-adapted leaves of 30-d-old seedlings of tomato that were grown under the HPS lamp and under the various LED light spectra (L1–L7). The results were plotted on a logarithmic scale from F_0_ (50 μs) to 1 s. The time points that were important for the calculations of OJIP test are marked: O—fluorescence intensity recorded at F_0_ (50 μs), K—at 300 μs, J—3 ms, I—30 ms, P—300 ms. (**A**) The induction curves of the relative variable fluorescence (Vt) of the tomato leaves that were obtained by the double normalisation of the fluorescent signal to F_0_ and F_M_. (**B**) The differential curves of ΔVt of the tomato leaves were obtained by subtracting the control value Vt (for HPS) from the values of the Vt for plants from lamps L1–L7. HPS—sodium lamp; L1—blue/red light; L2—blue/red light + far red; L3—blue/red light + UV; L4—white light supplemented with green; L5—white light supplemented with blue; L6—white light supplemented with red; L7—white light supplemented with blue/UV. HPS—L5—constant light 300 μmol m^−2^ s^−1^; L6–L7 light modulated (sunrise and sunset accomplished by gradually increasing/decreasing the light intensity, in the middle of the day the light intensity was increased to 700 μmol m^−2^ s^−1^ for 2 h; for details see chapter 3.1).

The condition of PSII in the dark-adapted state is described by several parameters, which were calculated from the OJIP test (Figure 3 and also the chlorophyll fluorescence transients in Figure 4). The area above the fluorescence induction curve from F_0_ to F_M_ (AREA parameter) is proportional to the size of the electron acceptors from PSII [34,35]. When the electron transport from the PSII reaction centre to the plastoquinones is blocked as a result of stress, this value is reduced. In the blue/red light variants L1–L3 (but also in L4), the AREA value decreased compared to both the HPS and to the white light variants, especially those with daily fluctuations of the light intensity—L6 and L7 (Figure 3A). This result indicates a decrease in the PSII electron acceptors in the blue/red light variants and in the white light that was supplemented with green. This was more or less in concert with a decrease in the Sm parameter (a normalised AREA, AREA/F_V_) and in the Q_A_ turnover number (N) (Figure 3B,C). In our experiment, the lowest Sm values were noted in the plants that were grown under L2 and L4; however, in all of the L1–L5 variants, these values were somewhat lower than in the HPS. In L6 and L7 (and partly in L3), the Sm values were comparable to the HPS. In a similar manner, the N values were lower in L1, L2, L4, and L5. Sm is regarded as being the energy that is required to close all of the PSII reaction centres, while N represents the time-dependent turnover number of Q_A_ [35].

The maximal PSII quantum yield (F_V_/F_M_) indicates the probability that a trapped photon will end up in the reaction centre and cause a photochemical event [35]. In our experimental setup, this parameter was significantly decreased only in the plants that were grown under L3 (blue/red+UV), compared to plants from HPS (Figure 3D). This indicates a PSII photoinhibition only in L3.

The relative variable fluorescence at step J (after 2 ms), which is represented by V_J_ calculated as (F_J_ − F_0_)/(F_M_ − F_0_), provides information about the number of closed RCs relative to the total number of PSII RCs [36]. In plants that were grown in the light combinations L1–L4, this value was clearly higher compared to both the plants from the HPS and to the plants from white light combinations (L5–L7) (Figure 3E). Collectively, these parameters revealed that in the dark-adapted plants, there was a bigger pool of closed PSII reaction centres in the variants with blue/red light and with the white light that was supplemented with green. However, it should be noted that this closure may represent either a PSII photoinhibition or a temporary Q_A_ decrease that is caused by an accumulation of reducing power in the stroma.

A comparison of the plants that were illuminated with LEDs to those under the HPS revealed no significant differences in the ABS/CSm with exception of L2 (increase) and L3 (decrease) (Figure 3F). When the absorption of light per PSII reaction centre (ABS/RC, Figure 3G) among the variants was compared with white light, there was a slight tendency to reduce the PSII antenna in the presence of UV. Such a tendency was, however, absent in the case of the blue/red variants. Considering the increased proportion of closed PSII RCs in these variants, it may suppose that additional supplementation with UV does not have any further effect on PSII.

In the dark-adapted plants, the probability of electron transport beyond QA (described by ET_0_/RC) was proportional to the amount of open PSII RCs and to the redox state of the PQ pool. In agreement with an increased V_J_, values of ET_0_/RC were reduced in variants L1–L4 compared to the HPS (Figure 3E,H). A similar tendency was also noted for the plants from the white light variants L5–L7. These results suggest that the HPS light ensures the lowest PSII excitation energy among the tested variants as well as the highest PQ pool oxidation in darkness.

Based on the ΔVt curves (Figure 4A,B), it is visible that the most pronounced change was found in the plants that were grown under the L4 light. All of the analysed steps (O–K, O–J, J–I, and I–P) had a higher course at all steps compared to the control lamp (HPS). The increase in the ΔV_O-K_ (in the conditions of L4, but also under L1 and L2) might be associated with the inactivation of the oxygen evolving complex (OEC) and/or the inhibition of electron transport on the donor or acceptor side of photosystem II [37]. ΔV_O-K_ also provides information about the grouping or connectivity—the relative position of the antenna complexes of different RCs in relation to each other [38]. A positive change in the course of a curve indicates greater distances between the PSII antennae and therefore a less efficient energy exchange [39,40]. The use of L1, L2, and L4 caused a significant increase in ΔV_O-J_. The O–J phase can be used as an indicator of a reduction of the acceptor side of PSII [41]. The L6 light caused a decrease in the last phase (I–P). ΔV**_I-P_** provides information about the electron flow to the end of the electron acceptors of PSI [42]. Generally, compared to the plants from the HPS, the closest course of the curves was noted for plants from the white lights L5–L7 (Figure 4A,B). The curves that characterised the plants from the blue/red variants and also from L4 were more distant/differentiated compared to those for the plants from the HPS.

**Figure 5:**
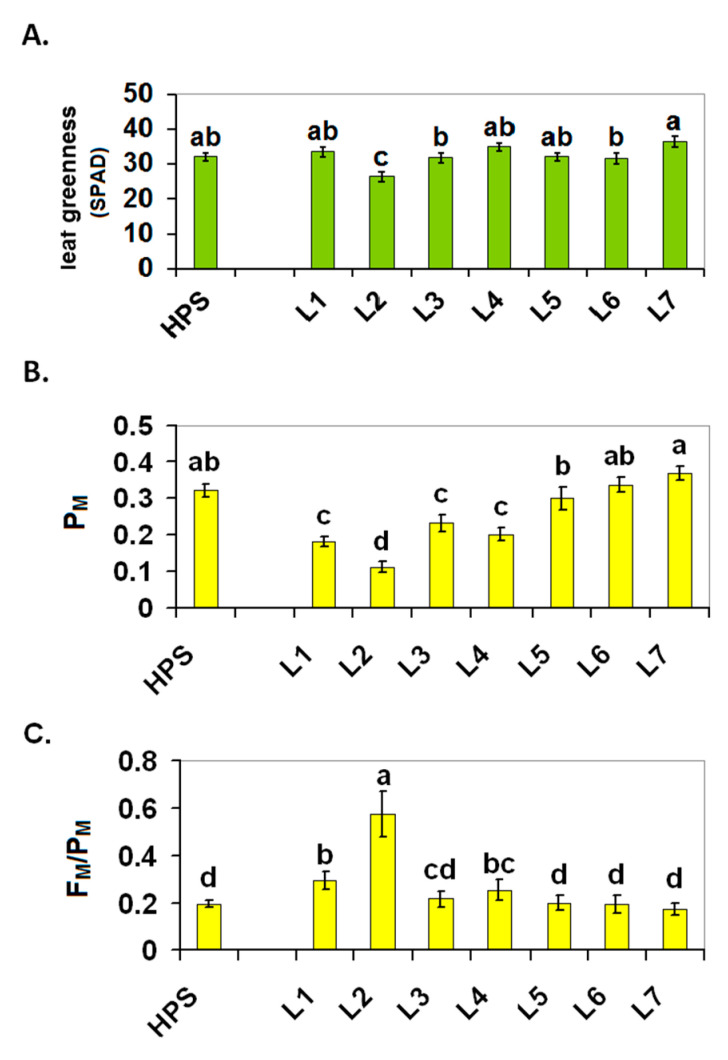
Leaf greenness in the SPAD values (**A**), the maximal redox change at PSI, P_M_ (**B**) and the ratio of F_M_/P_M_ (**C**) determined on the leaves of the 30-d-old tomato seedlings that were grown under the HPS lamp and under various LED light spectra (L1–L7). The values that represent the mean ± SD that are marked with the same letters are not significantly different according to the Duncan test (*p* ≤ 0.05). HPS—sodium lamp; L1—blue/red light; L2—blue/red light + far red; L3—blue/red light + UV; L4—white light supplemented with green; L5—white light supplemented with blue; L6—white light supplemented with red; L7—white light supplemented with blue/UV. HPS—L5—constant light 300 μmol m^−2^ s^−1^; L6–L7 light modulated (sunrise and sunset accomplished by gradually increasing/decreasing the light intensity, in the middle of the day the light intensity was increased to 700 μmol m^−2^ s^−1^ for 2 h; for details see chapter 3.1).

In our experiment, leaf greenness was also measured non-invasively using a chlorophyll meter and those measurements revealed only slight changes in the chlorophyll content in the leaves, although the plants from L2 (combination with FR light) were characterised by slightly less leaf greenness than the plants from under the other lamps (Figure 5A).

Insight into the functionality of PSI and PSII is provided by a so-called quenching analysis during the induction of photosynthesis under actinic illumination. The starting point for this analysis is the determination of the relative PSI and PSII proportions. An approximate measure of the proportion of PSII/PSI might be obtained by determining F_M_ and P_M_. At room temperature, F_M_ originates exclusively from PSII, while P_M_ provides information about the maximal redox change at PSI RC (P700), which is proportional to the number of active PSI units. The lower P_M_ values in variants L1–L4 suggest that these light conditions negatively affect PSI, which finally leads to a decrease in the number of active PSI units (Figure 5B). This is in line with the increased F_M_/P_M_ in these variants with the exception of L3. The strongest decrease in P_M_ and the highest ratio of F_M_/P_M_ (Figure 5C) was detected in L2, which indicates that a high proportion of FR selectively overexcites chlorophyll in the PSI reaction centres (P700). While a short-term FR excitation oxidises the PQ pool and P700, continuous illumination with FR may lead to an overreduction of P700 due to cyclic electron transport around PSI and/or to a charge recombination at PSI [4,43]. Such a situation may cause the generation of ROS from PSI and PSI damage. Hence, a probable mechanism of the acclimation of plants to be able to grow under FR may be by limiting the active PSI units.

**Figure 6:**
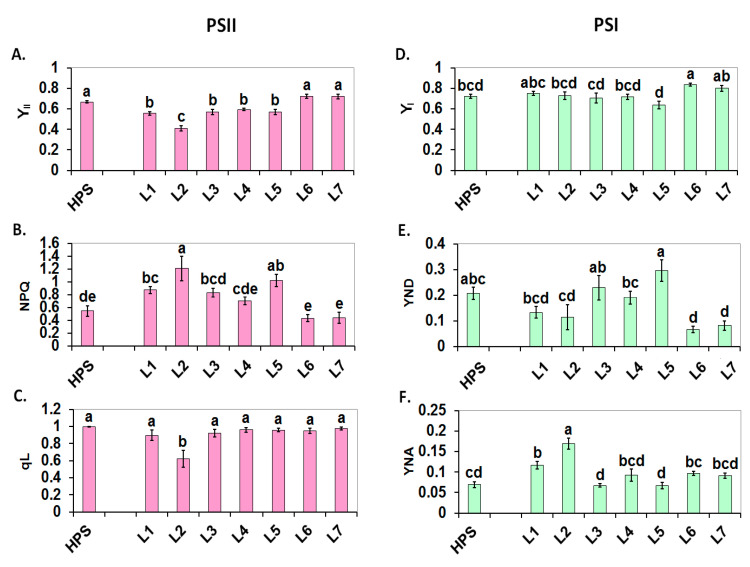
The functionality of the two photosystems under actinic illumination. The PSII parameters Y_II_, NPQ and *q*L are shown in (**A**–**C**), respectively. The PSI parameters Y_I_, YND, and YNA are shown in (**D**–**F**), respectively. The leaves of 30-d-old tomato seedlings that were grown under the HPS lamp and under various LED light spectra were measured (L1–L7). The values that represent the mean ± SD that are marked with the same letters are not significantly different according to the Duncan test (*p* ≤ 0.05). HPS—sodium lamp; L1—blue/red light; L2—blue/red light + far red; L3—blue/red light + UV; L4—white light supplemented with green; L5—white light supplemented with blue; L6—white light supplemented with red; L7—white light supplemented with blue/UV. HPS—L5—constant light 300 μmol m^−2^ s^−1^; L6–L7 light modulated (the sunrise and sunset accomplished by gradually increasing/decreasing the light intensity, in the middle of the day the light intensity was increased to 700 μmol m^−2^ s^−1^ for 2 h; for details see chapter 3.1).

The functioning of light-adapted PSII (Y_II_, the effective PS II quantum yield) was decreased in three of the variants of blue-red light (L1-L3) and in two variants of white light that was supplemented with green (L4) and with blue (L5) compared to the HPS lamps (Figure 6A). These results indicate that these light conditions are not optimal for the activity of PSII. The strongest decrease in Y_II_ occurred in the plants that were grown under FR (L2), which can be interpreted by the effects of FR on PSI and the PQ pool rather than by the direct effect of FR on PSII. It is noteworthy that the two variants of white light that were supplemented with high light (HL) at midday (L6, L7) were similarly as effective as the HPS.

Increased NPQ is indicative of the acidification of the thylakoid lumen and for an enhancement in the protective xanthophyll cycle [44]. The highest NPQ value was noted in L2. Because a high intensity of cyclic electron transport (CET) increases lumen acidification [45], this result supports the suggestion of the intensification of CET around PSI under FR (Figure 6B). A high ΔpH, in turn, can inhibit the cytochrome b_6_f activity which keeps PQ pool reduced [46]. In agreement with this view, there was a decrease in the proportion of open PSII reaction centres (*q*L) in L2 (Figure 6C) along with a reduced ET_0_/RC, which indicates a reduced Q_A_. Our results of red/blue illumination are in contrast to those described by Xiaoying et al. [24], where several beneficial effects of red/blue and red/blue/green on growth and photosynthesis have been found in comparison to white light. Among these differences were a higher rate of net photosynthesis and a higher PSII quantum efficiency. However, it has to be noted that the spectrum of “control” white light was characterized by the two biggest peaks in UV and yellow. This high contribution of UV portion might be responsible for some declined effects in controls.

In contrast to PSII, the effective PS I quantum yield (Y_I_) was fairly similar in all of the tested variants with the exception of L6, where it was enhanced (Figure 6D). Our data on photosystem efficiencies under blue/red are similar to the results of Yang et al. [27]. These authors noted that the best photosynthetic performance and the highest PSII and PSI quantum yields occur under white light, although the negative impact of blue/red irradiance was only minor. It was represented by a decline of ETRII at the highest light intensities of the rapid light curve and by a slight inefficiency to oxidize P700 by FR. Light variant L6 seems to be particularly suitable for the functioning of PSI as was visualised by a lower I point on the OJIP curve compared to the HPS (Figure 4A,B). However, both variants of white light that were supplemented with HL at midday, namely L6 and L7, were characterised by a reduced donor-side limitation of PSI (YND) (Figure 6E), which can be interpreted in terms of an efficient linear electron flow. Possibly, the short-term application of HL around midday caused some beneficial effects on the photosynthetic apparatus, among which could be a strong activation of Rubisco, an improved management of assimilates, stomatal opening, etc. In contrast, in L2 and to a lesser extent in L1, there was a significantly enhanced YNA (Figure 6F). This indicates an accumulation of photosynthetic reducing power at the acceptor side of PSI, which might be caused, for example, by an inefficient Calvin cycle and/or by an overreduction of the stroma.

The highest values of net photosynthesis (P_N_, nearly 15 µmol (CO_2_) m^−2^ s^−1^) were obtained for the plants that were grown under L4 (Table 1). This was accompanied by the highest transpiration intensity and stomatal conductance. The intracellular concentration of the CO_2_ values was comparable to those noted in plants from the other light combinations. The value of the P_N_/E (WUE) relationship, which provides information about the water use efficiency was one of the lowest in the plants that were grown under L4. Quite high P_N_ values were also obtained for the plants that were grown under the L2 lamp. In the remaining cases, the P_N_ values were comparable and fluctuated around 9 µmol (CO_2_) m^−^^2^ s^−^^1^. According to the literature, in studies on cherry tomatoes, Liu et al. [24] did not observe an increase in the P_N_ values under red/blue/green light compared to blue/red. However, the red/blue/green combination of spectral energy distribution was 3:3:1, which was different than those that were tested in our research in which green light was dominant compared to blue or green (L4, see picture with light spectra in M&M section). On the other hand, an explanation of the phenomenon of the increased intensity of photosynthesis under green light supplementation was proposed in the work of Sun et al. [18]. When studying spinach plants, the authors noted that the ^14^CO_2_ fixation under green light was less intense in the upper epidermal layer and uppermost palisade mesophyll compared to red and blue light, but that green light induced more fixation deeper in the leaf than red or blue. A possible mechanism of that phenomenon is based on the fact that the excitation of chlorophyll at the top of a leaf by blue and red light is higher than with green light, which may suggest the possibility that the top of a leaf has a greater capacity for non-photochemical quenching than the underlying tissue. Deeper in a leaf, where mainly green light is absorbed, non-photochemical quenching would not be as active. Taking all this into consideration, we can presume that large amounts of green light that possibly penetrate into the deeper layer of leaf tissues could also have been somehow connected to the increased CO_2_ assimilation in our experiment on tomato seedlings.

### 2.3. Plant Metabolic Profile

**Figure 7:**
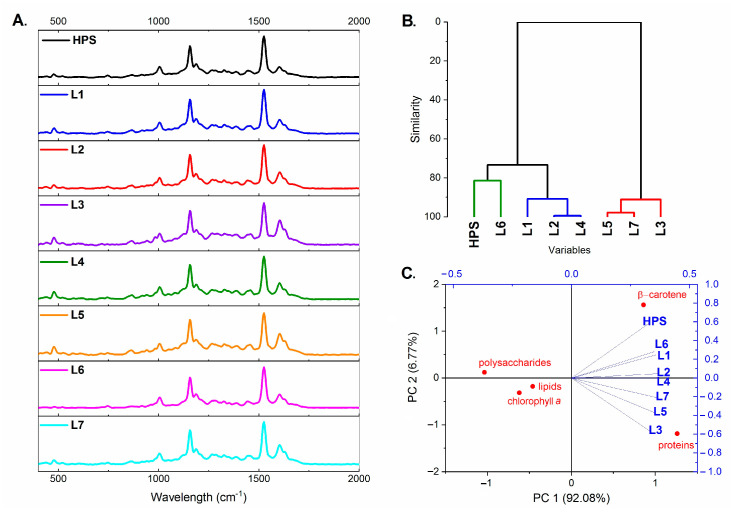
The Raman spectra for the tomato leaves that were grown under different light conditions (**A**). The hierarchical clustering analysis (HCA) from the entire range of the Raman spectra that revealed the similarities in the chemical composition of the tomato leaves that were grown under the different spectral composition of light (**B**). The principal component analysis (PCA) of the data of the tomato leaves from the Raman spectroscopy (**C**). This two-dimensional (2D) score plot presents the differences in the metabolic compositions. For the PCA analysis, the intensity of the Raman peaks was used at the appropriate wavelength for the functional groups. HPS—sodium lamp; L1—blue/red light; L2—blue/red light + far red; L3—blue/red light + UV; L4—white light supplemented with green; L5—white light supplemented with blue; L6—white light supplemented with red; L7—white light supplemented with blue/UV. HPS—L5—constant light 300 μmol m^−2^ s^−1^; L6–L7 light modulated (sunrise and sunset accomplished by gradually increasing/decreasing the light intensity, in the middle of the day the light intensity was increased to 700 μmol m^−2^ s^−1^ for 2 h; for details see chapter 3.1).

FT-Raman spectroscopy, used in our experiment for determination of plant metabolic profile, is a non-destructive and rapid analysis that can be used as a method in plant breeding and selection, plant phenotyping, nutrient analysis, and especially for detecting and imaging the photosynthetic pigment concentration. However, this method generally helps in detecting biotic and abiotic stresses in plants [47,48,49,50,51]. In our research, the Raman spectra were used to reveal the differences in the chemical composition of the tomato leaves that were grown under the different light conditions. The Raman spectra that were obtained for tomato leaves had bands for the photosynthetic pigments, the phenolic compounds, lipids, carbohydrates and proteins, regardless of the spectral composition of light. The spectra for all of the analysed leaves are presented in Figure 7A and the position of the peaks (Raman shift) and information about the vibrations are presented in Table 2.

Particularly intense peaks (carotenoids, region 1004/1155/1525 cm^−1^) were clearly visible for the leaves of plants that were grown under all lamps (Figure 7A). As for other peaks, a peak in the 476 cm^−1^ region (starch) was observed for the leaves that were grown under HPS, L1, L3, L4, L5, and L7 lamps, but it was slightly more visible for the leaves of the plants that were grown under the L6 lamp. A hierarchical clustering analysis (HCA) and principal component analysis (PCA) of the Raman spectrum revealed the similarities and differences of the chemical composition of the tomato leaves that were grown under the different spectral composition of light (Figure 7B,C). The cluster analysis (HCA) revealed that three homogeneous groups for chemical composition could be distinguished (Figure 7B). A similarity in the chemical composition was found between the leaves that were grown under the L5, L7, and L3 lamps (the first group), the second group was formed by the leaves that were grown under the L1, L2, and L4 lamps, while the third group was formed by the leaves that were grown under the HPS and L6 lamps (Figure 7B). The PCA analysis of the Raman data revealed differences in the content of proteins and β-carotene due to the spectral composition of light (a positive correlation). The highest levels of carotenoids in the tomato leaves were found in the plants that were grown under the HPS. The highest levels of the proteins were found in the plants that were grown under L3 and L5 light. There was a negative correlation between the light conditions and the content of chlorophyll *a*, polysaccharides, and lipids (Figure 7C), which means that the differences in the lighting had no effect on these compounds (or only had a very slight impact). In the case of chlorophyll however, more exact data were obtained by measurements using a chlorophyll meter (compare Figure 5A).

### 2.4. Balance of the Selected Plant Hormones (Auxins and Brassinosteroids)

**Figure 8:**
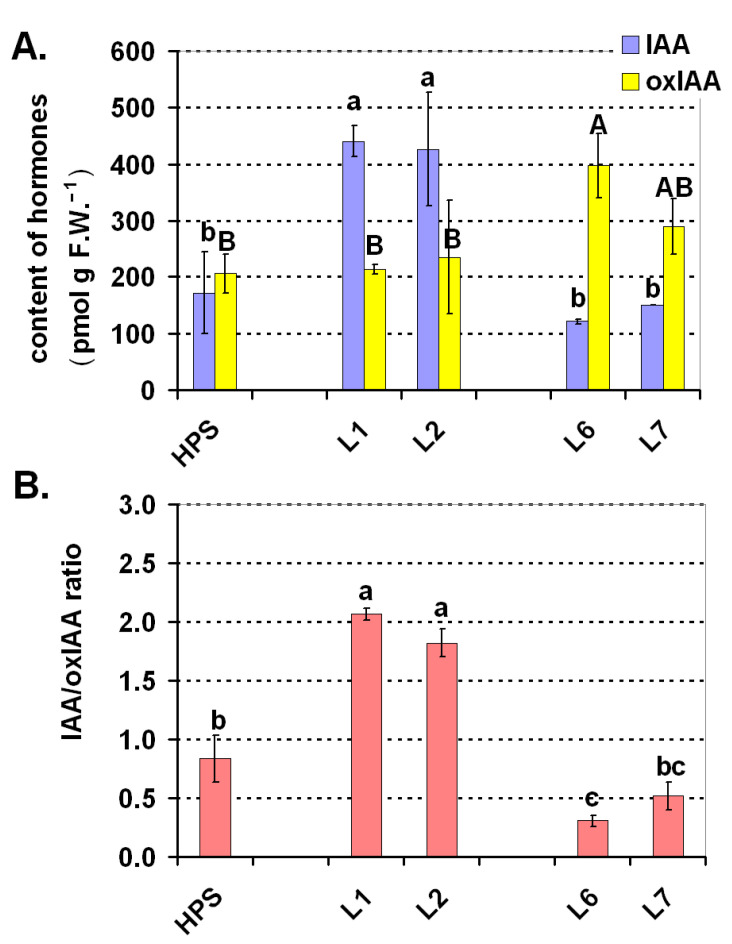
Balance of the active auxin indole-3-acetic acid (IAA) and its degradation product 2-oxindole-3-acetic acid (oxIAA) in the 30-d-old tomato seedlings that were grown under the HPS lamp and under the selected LED light spectra: blue/red variants (L1, L2) and variants of white light (L6, L7). (**A**) Concentration of IAA and oxIAA, (**B**) IAA/oxIAA ratio. Values (±SD) marked with the same letters are not significantly different according to the Duncan test (*p* ≤ 0.05) (in Figure A, lower case letters show the comparison for IAA and uppercase letters for oxIAA). HPS—sodium lamp; L1—blue/red light; L2—blue/red light + far red; L6—white light supplemented with red; L7—white light supplemented with blue/UV. HPS—L2—constant light 300 μmol m^−2^ s^−1^; L6–L7 light modulated (sunrise and sunset accomplished by gradually increasing/decreasing the light intensity, in the middle of the day the light intensity was increased to 700 μmol m^−2^ s^−1^ for 2 h; for details see chapter 3.1).

Two auxins were detected in the tomato leaves—the main active auxin (IAA) and its degradation product (oxIAA). The concentration of both IAA and oxIAA in the tomato 30-d-old seedlings was dependent on light conditions. Under the HPS, the concentration of IAA and oxIAA was on similar level (Figure 8A) and therefore the IAA/oxIAA ratio was close to 1 (Figure 8B). The situation under the LED variants with blue and red light (L1, L2) was different and the content of active IAA was two-fold higher than oxIAA (and than IAA under the HPS as well; Figure 8A). Once again, the opposite effect was observed for the plants that were grown under the LED variants of white light (L6, L7), in which oxIAA was dominant compared to IAA, which was also reflected in the low IAA/oxIAA ratio (about 0.5). OxIAA is a degradation product of IAA [56] and the enzymes that are responsible for this process are also present in cereals such as maize [57]. OxIAA is slightly active as an auxin and it has been proven in many studies that its content increases with an increase in IAA, which helps to maintain auxin homeostasis [56]. Our study seems to indicate that this conversion (IAA->oxIAA) might also be regulated by light. Namely, green light appeared to stimulate the process of IAA degradation into oxIAA. This was generally visible when the plants that were cultured under L1 and L6 were compared, because between the L1 and L6 spectra the only difference was the addition of green light (the ratio of blue and red was similar, as compared in Figure 10). When the plants from the HPS, L6, and L7, were compared to the plants from L1 and L2, it appeared that the blue/red variants most likely limited the conversion of IAA to oxIAA, thereby enabling the plants to accumulate more of the active auxin form—IAA. Of course, this hypothesis requires further studies. According to Liu et al. [58], light regulates the polar auxin transport in dark-grown tomato hypocotyls. An increase is only induced by red or blue light that is followed by darkness. Additionally, according to studies of Rubinstein [59], red light increases the sensitivity of tissues to the IAA hormone. On the other hand, it has long been known that green light retards growth [60]. According to these authors, the effects of green light generally oppose those that are directed by the red and blue wavebands and the green light sensory systems adjust the development and growth in conjunction with the red and blue sensors. Folta and Maruhnich [60] cited the early works of another author, Went (in book from 1957), who studied tomato plants under various light conditions. Seedlings that were grown under red plus blue light had more vegetative tissue than those that were grown under white light (primarily, red, blue, and green light). Went concluded that there was an “inhibitory effect of green light”. Our findings in some way seem to agree with those observations because the balance of auxins was altered in the direction of the inactive auxin oxIAA under L6 and L7 compared to L1 and L2. This seems, at least in part, to provide an explanation for those early observations. Although auxins are not the only players in regulating tomato growth, Higashide et al. [61] showed the inhibition of the growth of tomato seedlings by the auxin biosynthesis inhibitors. On the other hand, Almansa et al. [62] proved that there was a direct relationship between an increase of the total dry weight of tomato plants and an increase of the auxin concentration in tomato plants that were somewhat dependent on the light variations (UV, blue, red, and far red ratios). Interestingly, the absence of the red component reduced the IAA in many of the tomato cultivars even to 0. In our experiment, the active auxin (IAA) content was proportional to the accumulation of both the fresh and dry mass. A blue/red combination resulted in the highest accumulation of biomass, while the 30-d-old plants that were grown under the HPS and under L6 and L7 accumulated a significantly lower biomass (Figure 2E,F).

**Figure 9:**
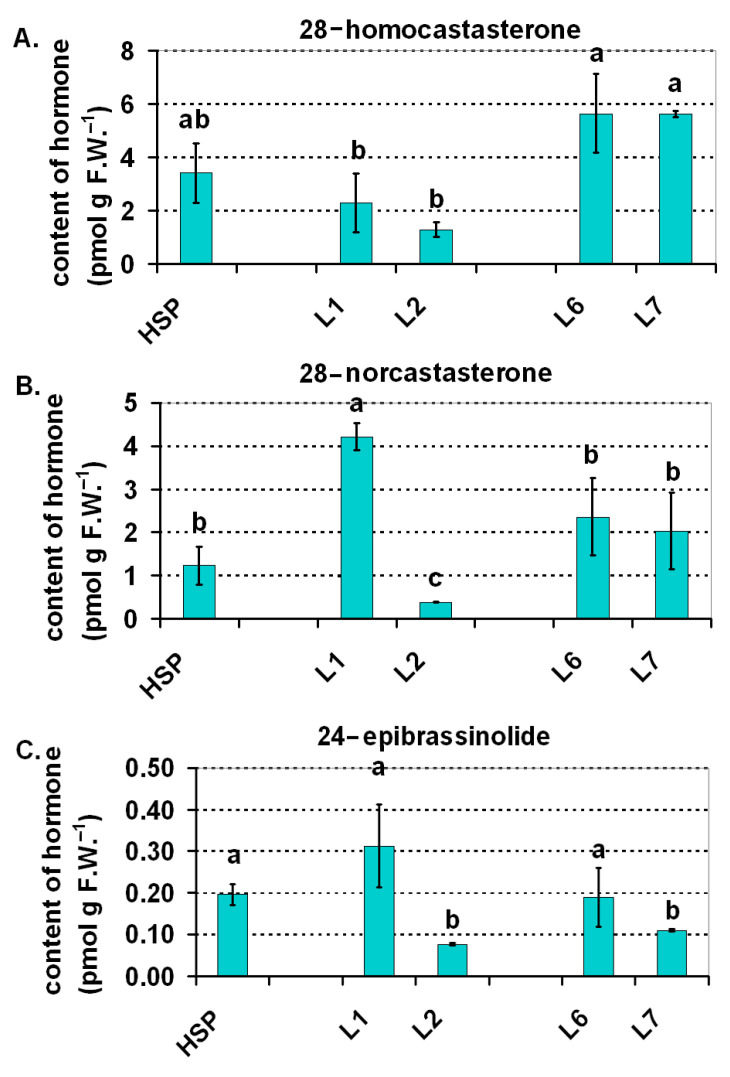
Contents of brassinosteroids in the 30-d-old tomato seedlings that were grown under the HPS lamp and under the selected LED light spectra: blue/red variants (L1, L2) and variants of white light (L6, L7). (**A**) Concentration of 28-homocastasterone, (**B**) concentration of 28-norcastasterone, (**C**) concentration of 24-epibrassinolide. Values (±SD) marked with the same letters are not significantly different according to the Duncan test (*p* ≤ 0.05). HPS—sodium lamp; L1—blue/red light; L2—blue/red light + far red; L6—white light supplemented with red; L7—white light supplemented with blue/UV. HPS—L2—constant light 300 μmol m^−2^ s^−1^; L6–L7 light modulated (sunrise and sunset accomplished by gradually in-creasing/decreasing the light intensity, in the middle of the day the light intensity was increased to 700 μmol m^−2^ s^−1^ for 2 h; for details see chapter 3.1).

Brassinosteroids (BR) are also plant growth-promoting hormones [63], although they generally have a multidirectional activity in plants, among others, as anti-stress protection agents [64]. For the light-regulated BR biosynthesis in plants, it was found that in rice both blue light and white light had a stimulating effect on BR biosynthesis [65]. An increase in the production of brassinosteroids (from the C_28_ biosynthetic pathway such as castasterone) was proven to be due to the blue-light mediated up-regulation of *CYP90A3* and *CYP90A4* (encoding the C-3 oxidases that is active in that pathway). On the other hand, the content of castasterone, which is considered to be the final product of the BR C_28_ pathway in rice [66], was two-folds lower under red light than under white or blue light. Far red very strongly limited the production of this hormone—more than four-folds compared to white light. Our results for tomato also revealed a modulating effect of light on the brassinosteroid content and profile in this species. The BR that was dominant in the tomato plants was 28-homocastasterone (BR from the C_29_ group) and accumulated the highest amount in the leaves under white light in L6 and L7 (Figure 9A). About a 30% lower content of this hormone was found under the HPS, about a half lower under blue/red (L1) and few times lower in the case of L2 (blue/red+FR).

Unlike 28-homocastasterone, the other BRs (28-norcastasterone from the C_27_ group and 24-epibrassinolide from the C_28_ group) were present in the highest amounts under blue/red light of L1 (Figure 9B,C). The addition of FR (L2) to the blue/red variant also limited the production of these BRs. L2 confirms the inhibiting effect of BR under FR, which was described in rice by Asahina et al. [65]. Under the HPS, the L6 and L7 content of 28-norcastasterone and 24-epibrassinolide in the plants was similar but was slightly lower than for blue/red light (L1). A few other BRs such as dolicholide, homodolicholide or 28-norbrassinolide were detected but only in trace amounts in the tomato leaves (data not shown).

In the context of measurements of photosynthetic efficiency that were taken, particularly those that revealed that the functioning of PSI was especially negatively affected under blue/red light that was supplemented with FR (thus in plants with a significantly lower content of BR), it is worth mentioning the regulatory role of these hormones in photosynthesis. BR have been found in chloroplasts [67], where they regulate the transcription of the genes whose products play a key role in the photosynthetic processes such as the *psaA* and *psaB* genes of photosystem I or the *psbA* and *psbD* genes of photosystem II [68]. The two proteins PsaA and PsaB form the heterodimer of the PSI reaction centre that binds the pairs of the primary electron donor and acceptor chlorophylls (P700 and A0, respectively) [69]. The photosynthetic efficiency under various LED light variants and its connection with brassinosteroids will require further investigation, in particular to determine the cause and consequence relation between these two variables.

The findings concerning auxin and brassinosteroid homeostasis under various lighting (especially under the LEDs) will also require further/deeper studies, especially for older tomato plants because the hormonal balance determines processes such as flowering, fruit setting, and final yield. The important role of these hormones was already independent of light conditions, described for tomato growth, yield, and quality ([70] and literature cited therein, [71,72,73]). Disturbances in the production and balance of hormones can be one of the crucial factors in the yield quantity/quality of tomatoes under the LEDs and studies of this issue might help to optimise LED lightening (in terms of wavelength proportion) in the indoor cultures of tomatoes.

Concluding remarks for all work are given in chapter 4.

## 3. Materials and Methods

### 3.1. Plant Material and Experimental Design

**Figure 10:**
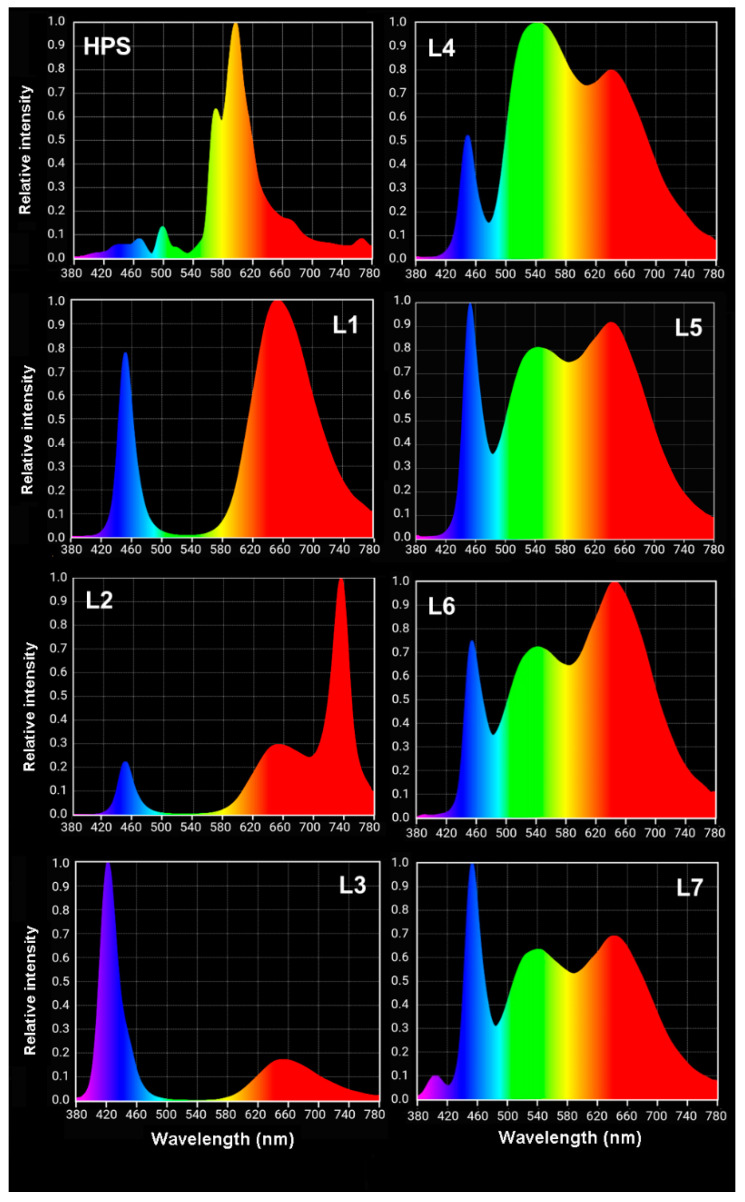
Visualising the light spectra that characterised the HPS and LEDs (L1–L7) that were used in the experiment. Details for the specific wave bands (peaks) as well as the blue/red and 440:680 ratios are given in Table S1, Supplementary Materials. HPS—sodium lamp; L1—blue/red light; L2—blue/red light + far red; L3—blue/red light + UV; L4—white light supplemented with green; L5—white light supplemented with blue; L6—white light supplemented with red; L7—white light supplemented with blue/UV. HPS—L5—constant light 300 μmol m^−2^ s^−1^; L6–L7 light modulated (sunrise and sunset accomplished by gradually increasing/decreasing the light intensity, in the middle of the day the light intensity was increased to 700 μmol m^−2^ s^−1^ for 2 h; for details see chapter 3.1).

Seeds of the tomato cultivar Beta (Polan, Cracow, Poland) were sown in pots with moist soil (200 seeds/pot size 40 cm × 15 cm × 15 cm) and placed into eight growth chambers with controlled temperature and light conditions (25 °C, 12 h photoperiod). Soil preparation: ‘Eko ziem universal soil’ (Jurków, Poland), soil from the cultivation plots at the University of Agriculture (Cracow, Poland), sand and ‘Substral Osmocote—a universal substrate’ (Scotts Poland sp. z o.o., Warsaw, Poland) were used at a ratio of 4:2:1:2. The light for each chamber was provided by either HPS or LED lamps. Philips SON-T AGRO 400 W HPS were installed in the first chamber, where the control plants of tomato were grown. In the other chambers, LED lamps produced by Plantalux (Konopnica, Poland) were installed, according to the order of Institute of Plant Physiology PAS: blue/red light (L1); blue/red light + far red (FR) (L2); blue/red light + UV (L3); white light that was supplemented with green (L4), and white light that was supplemented with blue (L5). For the HPS and chambers L1–L5, the light intensity was set at 300 μmol m^−2^ s^−1^ above the pots. In the last two chambers (L6 and L7), the LED light intensity was changing and imitated the sunrise, sunset, and moon. Chamber L6 had white light that was supplemented with red; the sunrise and sunset were accomplished by gradually (during 2 h) increasing the light intensity from 0 to 300 μmol m^−2^ s^−1^ (sunrise). At the end of a day, the light intensity was gradually decreased from 300 to 0 μmol m^−2^ s^−1^ (sunset, lasting 2 h). In the middle of the day, the light intensity was increased to 700 μmol m^−2^ s^−1^ (high light—HL) for 2 h. Chamber L7 had white light + blue/UV with the intensity modulated during the day in the same way as in L6. The light spectra for all of the lamps were measured using an Asensetek Lighting Passport Spectrometer (New Taipei City, Taiwan) and are presented in Figure 10. For comparison, the spectrum of natural sunlight was measured on a sunny day (morning) and is presented in Figure S1 (Supplementary Materials). Additionally, the detailed characteristics of all of the spectra of the lamps are given in Table S1 (Supplementary Materials).

In our research, the daily light integral (DLI) was calculated from the PAR data and was equal to 13 mol m^−2^ d^−1^ for lamps at an intensity of 300 μmol m^−2^ s^−1^ and to 30 mol m^−2^ d^−1^ for lamps, where for 2 h it was at an intensity of 700 μmol m^−2^ s^−1^.

The 12-d-old plants were moved to new pots; one plant per pot; pot size: 5 cm × 5 cm × 5 cm. The growth/morphological parameters were measured in 12-d-old seedlings and in 30-d-old plants. The leaf metabolic profile (measured using FT-Raman spectroscopy) and the content of auxins and brassinosteroids as well as chlorophyll *a* fluorescence, leaf gas exchange, and leaf greenness were measured in the 30-d-old plants. Additionally, the 12-d-old and 30-d-old plants were photographed.

### 3.2. Measurements and Observations

#### 3.2.1. Plant Growth Parameters

To assess the growth of the 12-d-old seedlings, the following growth parameters were collected: plant height, length of the part of plant above the cotyledons, first leaf length (including the petiole), stem diameter under cotyledons and, finally, the fresh mass of the aerial part of a plant. To assess the growth of the 30-d-old seedlings, the following growth parameters were collected: length of the part of a plant above the cotyledons, length and width of the fourth leaf, stem diameter under the cotyledons, and fresh and dry mass of the aerial part of a plant. Dry mass was measured after the plants were dried (72 h, 105 °C). Eighteen plants were measured (*n* = 18).

#### 3.2.2. Leaf Greenness

Leaf greenness (corresponding to the chlorophyll concentration) was analysed in 30-d-old plants using a non-invasive method using a chlorophyll meter (SPAD 502; Konica Minolta, Tokyo, Japan). Measurements were performed in the middle part of a fully developed leaf (the fourth leaf). Measurement was made in three technical replications per each leaf and then the average value was calculated for each leaf. Ten leaves (taken from 10 different plants) were measured (*n* = 10).

### 3.3. Chlorophyll a Fluorescence Measurements

#### 3.3.1. PSII Photochemistry Measured Using a Plant Efficiency Analyser

Chlorophyll *a* fluorescence was measured using a Plant Efficiency Analyser (PEA, Hansatech Ltd., King’s Lynn, UK) after the leaves had been adapted to the dark. The technical details of the measurements are described in Skoczowski et al. [74]. The following parameters were extracted/calculated based on the fluorescence curve (OJIP test): AREA (area above the fluorescence induction curve), Sm (normalised area), N (time-dependent turnover number of Q_A_), F_V_/F_M_ (maximum quantum yield of PSII), V_J_ (relative variable fluorescence at step J [after 2 ms]), ABS/CSm, ABS/RC and ET_0_/RC (where CSm—sample cross section; RC—reactive centre). More detailed equations for all of the parameters are given in Strasser et al. [75]. The physiological meanings of the parameters are additionally described in the chapter “Results and Discussion”. The fluorescence was measured in 10–12 replicates for each treatment (HPS, L1-L7). A biological replicate was one leaf (a fully developed leaf—the fourth leaf) of an individual plant. Additionally, the OJIP curves were prepared based on Bąba et al. [76], Kalaji et al. [36,77,78], and Strasser et al. [42]. OriginLab Software was used to perform the analyses and to draw the charts.

#### 3.3.2. PSI and PSII Photochemistry Measured Using a Dual-PAM

The PSI and PSII photochemistry were analysed using a Dual-PAM (Heinz Walz Gmbh, Effeltrich, Germany). Before they were measured, the leaves were dark adapted for 20 min to permit the fast fluorescence quenching mechanisms to relax. The PSII parameters were determined from the changes in chlorophyll fluorescence during the standard induction curve under red actinic illumination of 219 µmol PPFD m^2^/s. The effective PS II quantum yield (Y_II_) was calculated according to Genty et al. [79] and the coefficient *q*L, which describes the fraction of open PSII reaction centres, was calculated according to Kramer et al. [80]. Non-photochemical quenching (NPQ) was calculated according to Bilger and Björkman [44].

The PSI parameters were assessed from the absorbance changes at 830 and 875 nm according to Klughammer and Schreiber [81]. The photochemical quantum yield of PS I (YI) was calculated from the complementary PSI quantum yields, namely, the non-photochemical energy dissipation Y(ND) and Y(NA): Y(I) = 1-Y(ND)-Y(NA). Y(ND) represents the fraction of the overall P700 that has oxidised and is calculated from the fraction of the overall P700 that is reduced (P700 red.): Y(ND) = 1-P700 red, whereas Y(NA) represents the fraction of the P700 centres that cannot be oxidised with a saturation pulse and is calculated from the equation: Y(NA) = (P_M_-P_M_′)/P_M_, where P_M_ and P_M_’ represent the maximal change of the P700 signal after the saturation pulse is applied in a dark-adapted state and light state, respectively.

### 3.4. Leaf Gas Exchange

Gas exchange was measured using an LCpro-SD infrared gas analyser (ADC BioScientific Ltd., Hoddesdon, UK) with automatic control of the measurement conditions. The parameters that were measured included: the photosynthetic rate (P_N_), which provides information about CO_2_ assimilation; leaf stomatal conductance (g_s_) as well as the intercellular concentration of CO_2_ (C_i_). The water use efficiency index (WUE) was calculated based on the P_N_/E relationship. The conditions for the measurements: carbon dioxide concentration 360 μmol mol^−1^ air, temperature 25°C, measured under a given light: HPS, L1–L7. The middle part of the best-developed leaf (usually the fourth) was measured in eight biological replicates (replicate = one leaf from different plants).

### 3.5. FT-Raman Studies of the Metabolic Profile

The Raman spectra of fresh tomato leaves were recorded using a Nicolet NXR 9650 FT-Raman Spectrometer (Thermo Scientific, Walthman, MA, USA) equipped with an Nd:YAG laser (1064 nm) and an InGaAs detector. The measurements were taken in the range of 400 to 2000 cm^−1^ at a laser power of 0.5 W (64 scans per spectrum). The diameter of the unfocused laser beam was approximately 50 μm and the spectral resolution was 8 cm^−1^. The Raman spectra were processed using the Omnic/Thermo Scientific software (Thermo Scientific, Walthman, MA, USA).

The principal components analysis (PCA) and hierarchical clustering analysis (HCA) were performed in order to obtain information about any variations in the chemical composition of the leaves that were dependent on the type of lamp that was used. The Euclidean distance was used in the HCA analysis. The distance between similar groups was measured using the Ward algorithm. PCA is a non-parametric method for obtaining information about the similarities and differences between samples. This method was used for all of the measurement ranges. The statistical and Raman spectra were created using OriginLab 2020 software (OriginLab Corporation, Northampton, MA, USA).

The positions of the Raman peaks for the analysed tomato leaves along with a description of the vibrations corresponding with the respective functional groups are given in Table 1 [52,53,54,55].

### 3.6. Analysis of the Phytohormones

#### 3.6.1. Auxins

Extraction of indole-3-acetic acid (IAA) and 2-oxindole-3-acetic acid (oxIAA) was performed as described in detail in Pěnčík at al. [82]. Briefly, tomato leaf samples (10 milligrams of fresh weight) were homogenized and extracted in 1 mL of 50 mM sodium phosphate buffer (pH 7.0) with the addition of internal standards: [indole-^13^C_6_]IAA and [indole-^13^C_6_]oxIAA. The samples were incubated at 4 °C with continuous shaking and then centrifuged (15 min, 23,000× *g* at 4 °C). Supernatants were then acidified with 1 M HCl to pH 2.7 and purified by solid phase extraction (SPE) using C8 columns (Bond Elut, 500 mg, 3 mL; Varian). After evaporation under reduced pressure, samples were analysed using HPLC system 1260 Infinity II (Agilent Technologies, Santa Clara, CA, USA) equipped with Kinetex C18 column (50 mm × 2.1 mm, 1.7 µm; Phenomenex) and linked to 6495 Triple Quad detector (Agilent Technologies, Santa Clara, CA, USA) following the methodology described in Novák et al. [83].

#### 3.6.2. Brassinosteroids

Brassinosteroids (BR) were extracted and analysed as described in Oklestkova et al. [84]. Briefly, samples of the plant material were powdered in liquid N_2_ and then mixed with 80% methanol. Deuterium-labelled internal standards of BR were added (25 pmol/sample, Olchemim s.r.o., Olomouc, Czech Republic). After centrifugation, the obtained supernatant was passed through Discovery DPA-6S columns (Supelco, Bellefonte, PA, USA) and immunoaffinity (IA) columns (Laboratory of Growth Regulation, Olomouc, Czech Republic). The BR were eluted from the IA columns using cold 100% methanol, samples were dried and again resuspended in 40 μL of methanol in order to measure the hormones on a UHPLC using a tandem mass spectrometer (UHPLC-MS/MS) with an ACQUITY UPLC^®^ I-Class System (Waters, Milford, MA, USA) and a Xevo™ TQ-S MS triple quadrupole mass spectrometer (Waters MS Technologies, Manchester, UK). The detailed conditions are given in Tarkowská et al. [85] or Oklestkova et al. [84]. The analyses were performed in three repetitions and each repetition included about 50 mg of fresh weight fourth leaves.

## 4. Concluding Remarks

Plant growth (morphological parameters) under LED light was only partly correlated with the photosynthetic efficiency of PSI and PSII or net photosynthesis and therefore the measurements of photosynthesis cannot explain all of the effects of the various LED light combinations on tomato growth. As is shown in Figure 1 (and especially for the older plants in Figure S2), there were some morphological/growth differences between the plants that were grown under the HPS and the plants that were cultured under all of the tested LED lights but the lack of (or poor) correlation between plant growth and photosynthesis only confirmed the impact of various light conditions on other processes, such as hormonal homeostasis. Compared to the plants that were grown under the HPS, the auxin balance was altered in the plants that were grown under various LED lights in the direction of a higher accumulation of the active IAA under the blue/red variants and a higher accumulation of the auxin degradation form (oxIAA) under white light (L6, L7). The content of brassinosteroids was also light dependent, and the dominant BR (28-homocastasterone) accumulated in the highest amounts under the LED-generated white light while FR reduced the BR content in the leaves. A further and deeper investigation into the impact of LED light on tomato hormonal homeostasis seems to be advisable because in older tomato plants, it might determine processes such as flowering, fruit setting, and final yield. Interestingly, the FT-Raman studies showed that the general metabolic profile of the leaves, which have the main metabolic components such as proteins, carbohydrates, or lipids, was especially similar in two groups of plants—those that were grown under the HPS and those that were grown under white light with a changing light intensity over the duration of a day (L6). As for photosynthesis, this work provides important theoretical data. By comparing several variants of the light spectrum, it was revealed that illumination with blue/red LEDs as the main components had a negative effect on the functioning of PSII compared to the white light and HPS light. Additionally, the severity of the effect of blue/red illumination on PSII depended on the relative proportion of blue and red and was the worst under the highest proportion of blue. The functioning of PSI was especially negatively affected only under blue/red that was supplemented with FR. It is worth mentioning that the negative effect of blue/red illumination was compromised by the supplementation with green for the intensity of the net photosynthesis. Moreover, a comparison of a few of the white light variants revealed that the short-term application of high irradiance around midday evoked some beneficial effects on the functioning of the photosynthetic electron transport. Taken together, based on the analyses of the majority of the photosynthetic parameters, it can be stated that compared to the HPS, the LED-generated white light with daily variations in intensity (particularly variant L6) was more beneficial than the blue/red variants.

## Figures and Tables

**Table 1:** Gas exchange in the 30-d-old seedlings of the tomatoes that were grown under the HPS lamp and under various LED light spectra (L1–L7). The percentage changes compared to the values that were obtained under HPS (100%) are given in the bracket. P_N_—net photosynthesis intensity, g_s_—stomatal conductance, C_i_—intracellular concentration of CO_2_, WUE—photosynthetic ratio of water use. The values (±SD) that are marked with the same letters are not significantly different according to the Duncan test (*p* ≤ 0.05). HPS—sodium lamp; L1—blue/red light; L2—blue/red light + far red; L3—blue/red light + UV; L4—white light supplemented with green; L5—white light supplemented with blue; L6—white light supplemented with red; L7—white light supplemented with blue/UV. HPS—L5—constant light 300 μmol m^−2^ s^−1^; L6–L7 light modulated (the sunrise and sunset accomplished by gradually increasing/decreasing the light intensity, in the middle of the day the light intensity was increased to 700 μmol m^−2^ s^−1^ for 2 h; for details see chapter 3.1).

Light Sources	P_N_ [µmol (CO_2_) m^−2^ s^−1^]	g_s_ [mol (H_2_O) m^−2^ s^−1^]	C_i_ [µmol (CO_2_) mol (air)^−1^]	WUE [µmol (CO_2_) mmol^−1^ (H_2_O)]
HPS	9.2 ± 2.4 ^(100%) bc^	0.18 ± 0.05 ^(100%) b^	226 ± 17 ^(100%) c^	3.3 ± 0.4 ^(100%) ab^
L1	8.8 ± 2.3 ^(−5%) c^	0.21 ± 0.09 ^(+20%) b^	254 ± 22 ^(+12%) b^	3.0 ± 0.7 ^(−10%) abc^
L2	12.5 ± 2.1 ^(+35%) ab^	0.26 ± 0.07 ^(+47%) b^	238 ± 17 ^(+5%) bc^	3.6 ± 0.5 ^(+7%) a^
L3	9.9 ±1.4 ^(+8%) bc^	0.28 ± 0.13 ^(+58%) b^	246 ± 23 ^(+9%) bc^	3.1 ± 0.7 ^(−8%) abc^
L4	14.8 ± 5.5 ^(+60%)a^	0.49 ± 0.29 ^(+178%) a^	246 ± 12 ^(+9%) bc^	2.6 ± 0.3 ^(−23%) cd^
L5	9.0 ± 3.9 ^(−2%) c^	0.23 ± 0.06 ^(+28%) b^	253 ± 31 ^(+12%) b^	2.7 ± 1.0 ^(−18%) bcd^
L6	8.7 ± 1.5 ^(−5%) c^	0.18 ± 0.03 ^(−1%) b^	248 ± 14 ^(+10%) b^	3.3 ± 0.3 ^(−2%) ab^
L7	9.0 ± 3.5 ^(−3%) c^	0.31 ± 0.13 ^(+73%) b^	280 ±16 ^(+24%) a^	2.3 ± 0.6 ^(−32%) d^

**Table 2:** The positions of the Raman peaks for the analysed tomato leaves along with a description of the vibrations corresponding to the respective functional groups [52,53,54,55].

Peak’s Position (Raman Shift, cm^−1^)	Vibrations
476	Carbohydrates (starch)
740/1263/1324/1386	Chlorophyll *a*
865	Pectin compounds
865/916/943	Polysaccharides
1455/1606	Proteins
1004/1155/1525	Carotenoids
1186	β-carotene
1286/1455	Lipids
1440/1606	Flavonoid compounds
1263/1625	Lipids (unsaturated fatty acids)

## Data Availability

Not applicable.

## Supplementary Materials

The following are available online at https://www.mdpi.com/article/10.3390/ijms222111517/s1, Figure S1: Spectrum of daily light measured outdoor (May 2021, Krakow, Poland), Figure S2: Architecture of the upper parts of exemplary 30-d-old seedlings of tomatoes that were cultured under the HPS lamp and under various LED light spectra (L1–L7), Table S1: Spectral composition of the light that was generated by specific lamps (HPS, L1–L7), for which the details of the specific peak parameters (maximum, width, area) were calculated. Table is complementary to Figure 10.
