# Supplementary material for: Insight into Details of the Photosynthetic Light Reactions and Selected Metabolic Changes in Tomato Seedlings Growing under Various Light Spectra
Source: Int J Mol Sci. 2021 Oct 26;22(21):11517. doi: 10.3390/ijms222111517 (PMC8584210; doi:10.3390/ijms222111517)
Supplement: Supplementary file 1 [file ijms-22-11517-s001.zip › Table S1.pdf]

Table S1. Spectral composition of the light that was generated by specific lamps (HPS, L1-L7), for which the details of the specific peak parameters (maximum, width, area) were calculated. Table is complementary to Figure 10. *HPS – sodium lamp; L1 - blue/red light; L2 - blue/red light + far red; L3 - blue/red light + UV; L4 - white light that was supplemented with green; L5 - white light that was supplemented with blue; L6 - white light that was supplemented with red; L7 - white light supplemented with blue/UV. HPS - L5 – constant light 300 µmol m*$^{-2}$ *s*$^{-1}$*; L6 - L7 light modulated (the sunrise and sunset accomplished by gradually increasing/decreasing the light intensity, in the middle of the day the light intensity was increased to 700 µmol m*$^{-2}$ *s*$^{-1}$ *for 2h; for details see chapter 3.1).*

Colours of Light	Peak Parameters	Spectral Composition of the Light							
		HPS	L1	L2	L3	L4	L5	L6	L7
UV	Maximum				400				401
	Width	-	-	-	3.4	-	-	-	25
	Area				100				266
BLUE	Maximum	437	451	449	432	447	453	453	452
	Width	45	23	26	31	26	26	24	23
	Area	193	753	439	2106	505	729	533	2266
	Maximum	465							
	Width	16				-			
	Area	85							
GREEN	Maximum	497				515	547	524	521
	Width	232				43	100	89	98
	Area	206				1058	2326	2031	6418
	Maximum					555			
	Width	-				80		-	
	Area			-		3842			
YELLOW	Maximum	567							
	Width	15							
	Area	484							
ORANGE	Maximum	597					-		
	Width	40							
	Area	2910							
RED	Maximum	641	649	647	667	646	645	646	645
	Width	35	90	80	98	100	100	100	100
	Area	337	3783	1880	1643	3256	2705	3990	9614
	Maximum	668							
	Width	64				-			
	Area	619							
FAR-RED	Maximum			734					
	Width	-		45			-		
	Area			1205					
**BLUE:RED**	**Ratio of the peak area**	**0.3**	**0.2**	**0.2**	**1.3**	**0.1**	**0.3**	**0.1**	**0.2**
**BLUE:RED**	**Ratio of 440:680 nm**	**0.4**	**0.5**	**0.6**	**3.6**	**0.7**	**0.6**	**0.4**	**0.9**
