# Supplementary figures and images for: Insight into Details of the Photosynthetic Light Reactions and Selected Metabolic Changes in Tomato Seedlings Growing under Various Light Spectra
Source: Int J Mol Sci. 2021 Oct 26;22(21):11517. doi: 10.3390/ijms222111517 (PMC8584210; doi:10.3390/ijms222111517)

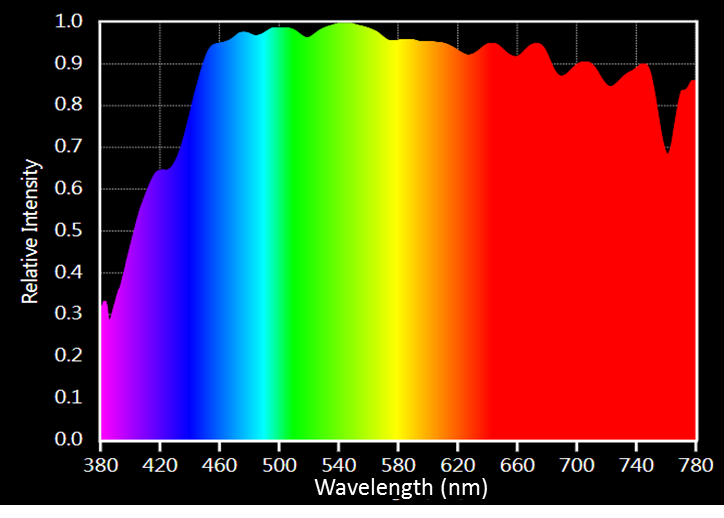

Supplement: Supplementary file 1 [file ijms-22-11517-s001.zip › Figure S1.tif]

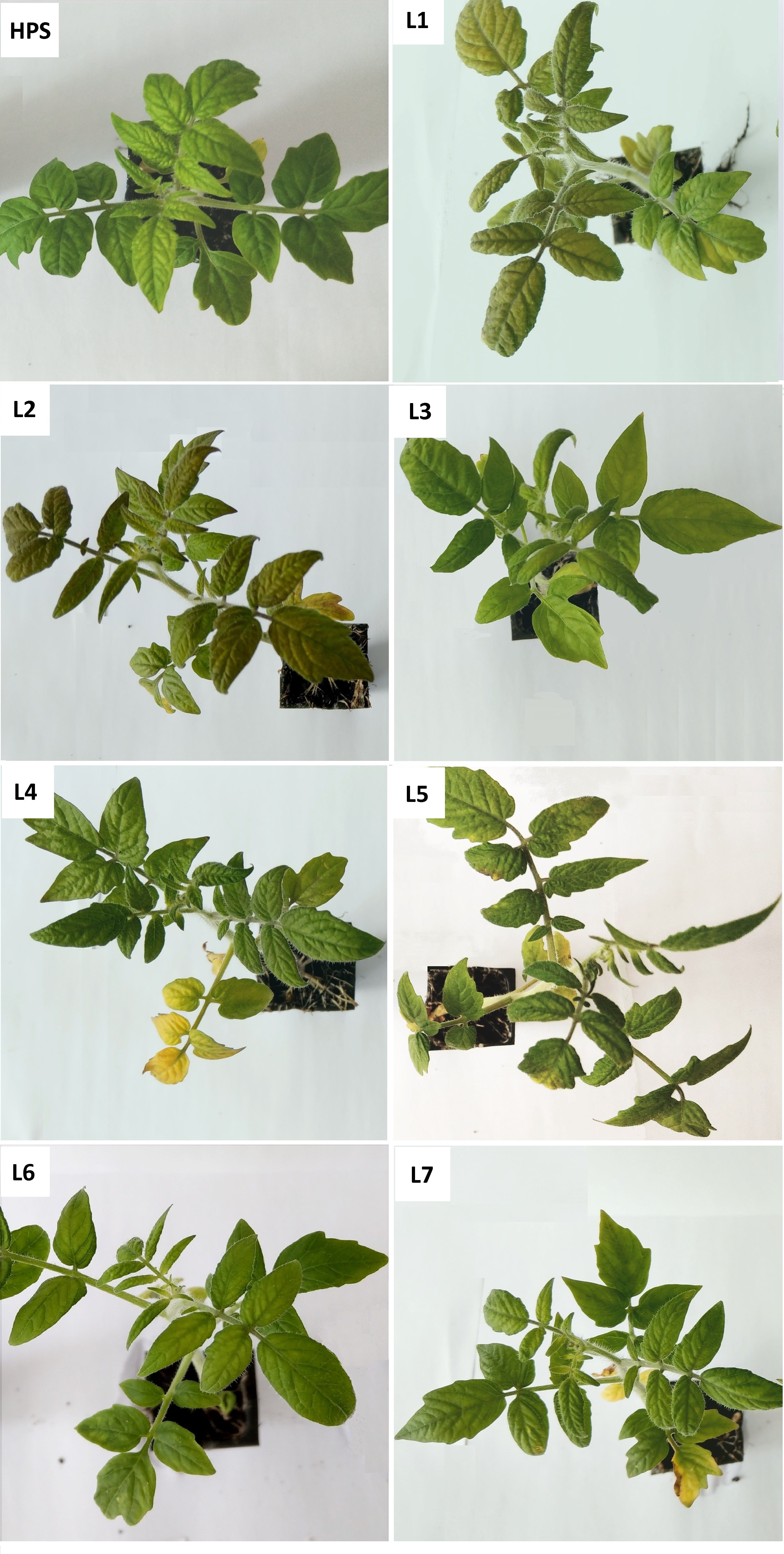

Supplement: Supplementary file 1 [file ijms-22-11517-s001.zip › Figure S2.jpg]
